# Mechanistic insights into N_2_ activation by RhCo_3_*via* d–d orbital coupling

**DOI:** 10.1039/d5ra06945a

**Published:** 2025-11-06

**Authors:** JingJing Wu, HaiXiong Shi, YongCheng Wang

**Affiliations:** a School of Chemical Engineering, Lanzhou University of Arts and Science Lanzhou 730010 China; b College of Chemistry and Chemical Engineering, Northwest Normal University LanZhou 730070 China 1001323@luas.edu.cn w278693923@163.com

## Abstract

The synthesis of ammonia (NH_3_) from nitrogen (N_2_) under mild conditions is a great challenge, in which the electron-donating ability of the catalyst is the key for N_2_ activation. In this work, the above process was studied using quantum chemical calculations using the density functional method. The results show that Rh and Co exhibit unsaturated d-electron configurations, with d–d orbital coupling occurring within the RhCo_3_ metal cluster, resulting in bimetallic synergy and spin effects. The Rh atom serves as an electron modulation center and active site for reactant activation, while the Co metal synergistically enhances electron back-donation effects. The RhCo_3_ cluster exhibits different adsorption and kinetic behaviors across the triplet, quintet, and septet potential energy surfaces, among which the septet state shows the most favorable catalytic performance. Energy span (δ*E*) model analysis further indicates that, at low and moderate temperatures, the N_2_ adsorption is the key factor governing the catalytic activity of RhCo_3_. At 298 K, the reaction displays a δ*E* value of 2.03 eV, and the catalytic activity increases with the temperature. However, at higher temperatures, the NH_3_ desorption becomes the rate-determining process, shifting the turnover-determining transition state beyond the turnover-determining intermediate, and the energy span increases to 2.95 eV. These findings elucidate the temperature-dependent catalytic mechanism of RhCo_3_ and provide theoretical insights for the rational design of efficient bimetallic catalysts for ammonia synthesis.

## Abstract 2

The RhCo_3_ catalyst exhibits bimetallic synergy and spin effects *via* Rh–Co d–d orbital coupling, facilitating efficient N_2_ activation for ammonia synthesis in the septet state.

## Graphical abstract

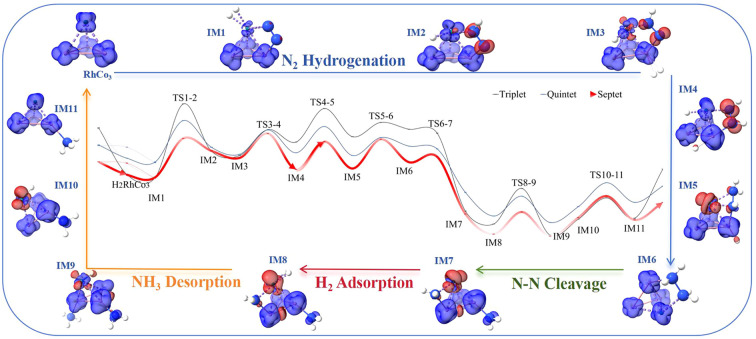

## 1. Introduction

Ammonia (NH_3_) is a crucial raw material used in the fertilizer, pharmaceutical, and chemical industries.^1,2^ Owing to its high hydrogen content, NH_3_ holds promise as a carbon-free energy storage fuel and is increasingly becoming a valuable resource in future energy systems.^3–5^ The low electron affinity (−1.8 eV), nonpolar nature, and high ionization energy (15.58 eV) of the nitrogen molecule make its activation challenging.^6^ Therefore, this process requires high-temperature and high-pressure conditions, leading to significant CO_2_ emissions and substantial energy consumption. In fact, the energy-intensive nature of ammonia production (primarily *via* the Haber–Bosch process) stimulates further research into more sustainable and efficient alternatives. The electron-donating ability of the catalyst has been shown to be crucial for the activation of N_2_. The development of a highly efficient catalyst for the nitrogen cycle could result in substantial energy savings and reduced consumption in the ammonia synthesis industry.^7^

Multimetallic cluster catalysts have the potential to achieve high catalytic activity,^8–11^ thanks to the enhanced synergy among multiple metals. The metal centers of these catalysts increase the electron-donating ability to the π* orbitals of N_2_ antibonds,^12^ thereby weakening the N≡N bond and facilitating the ammonia synthesis.^13,14^ This synergy results in a superior catalytic performance compared to single-metal catalysts;^15^ transition metals such as rhodium (Rh) and cobalt (Co), along with Fe and Ru, exhibit high activity in activating N_2_, as demonstrated by their volcano-type curves.^16,17^ However, Rh-based catalysts have several significant drawbacks, including high cost and poor stability, which limit their broad application.

The partially filled d orbitals of transition metals such as Rh and Co provide unique electronic modulation abilities, making them highly effective catalytic centers for small-molecule activation.^18^ Recent theoretical and experimental studies have shown that spin-related properties can influence the thermodynamics and kinetics of catalytic reactions.^19–21^ While Rh and Co are located in different rows of the periodic table, they both belong to group VIIIB, and their similar d-orbital electronic structures may induce d–d orbital interactions between them, enhancing their electron exchange and transfer properties with adsorbed species or intermediates.^22^ Furthermore, electron spin effects are crucial in regulating catalytic reactions; the incorporation of Co atoms into Rh active sites can create a bimetallic synergy, enhancing the spin effects.^23–25^ Thus, the development of RhCo_3_ catalysts, harnessing d–d orbital coupling to enhance spin interactions, represents a promising strategy for activating N_2_-based reactions. Nevertheless, the mechanistic interplay between synergistic and spin effects in bimetallic transition metal catalysts remains poorly understood. In this work, we employ quantum chemical calculations and a d–d orbital coupling strategy to explore the cooperative spin effects of isolated RhCo_3_ clusters in the N_2_ to NH_3_ conversion, focusing on the modulation of electron spins. Moreover, we apply the energy span model proposed by Kozuch to calculate the turnover frequency (TOF), establishing a framework for optimizing the performance of bimetallic catalysts.

## 2. Computational methods and theoretical background

### 2.1 Molecular geometry optimization

All calculations were carried out using the Gaussian 16 program.^26^ Density functional theory (DFT)^27^ calculations with the B3LYP functional^28^ were performed using the SDD pseudopotential basis set^29^ for the Co and Rh transition metals, while the 6-311++G(2df, 2pd) basis set was employed for the H and N atoms. The molecular geometric structures of all species within the reaction system, including reactants, intermediates, transition states, and products, were optimized and confirmed by frequency analysis. The thermodynamic properties, zero-point energies, and vibrational frequencies of the stable structures were then determined. The energies of reactants, intermediates, and products were confirmed as local minima (with no imaginary frequencies), while transition states exhibited unique imaginary vibrational frequencies. The conversion from transition states to reactants and products was analyzed using the intrinsic reaction coordinate (IRC) method to confirm the structural integrity. Wavefunction analysis of the reaction system was conducted using the Multiwfn package.^30^ The bond dissociation process was studied through electrostatic potential (ESP) theory,^31^ Mayer bond order analysis,^32^ and the electron localization function (ELF) method.^33,34^

### 2.2 Calculation of adsorption and dissociation energies

The adsorption and dissociation energies (*E*_ads_(X) and *E*_diss_(Y), respectively) reflect the interaction strength of adsorbates on catalysts and the energy required for their dissociation, respectively. They were calculated using the following equations:^35^

1 *E*_ads_(X) = *E*_X/catalyst_ − (*E*_catalyst_ + *E*_X_)

2 *E*_diss_(Y) = *E*_catalyst_ + *E*_Y_ − *E*_im_

where *E*_X/catalyst_ and *E*_catalyst_ are the energy of the adsorption complex and isolated catalyst molecule, respectively; moreover, *E*_X_ and *E*_Y_ represent the energies of the gas-phase adsorbed reactant X and dissociated product Y molecules, respectively. *E*_im_ is the energy of the pre-dissociation reaction intermediate (*e.g.*, an intact ammonia molecule adsorbed on the catalyst surface).

### 2.3 Calculation of TOF

The TOF reflects the activity of a catalyst, and was calculated based on the energy span model, as described by the following equations:^36,37^3
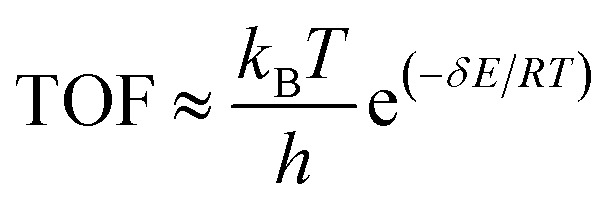
4
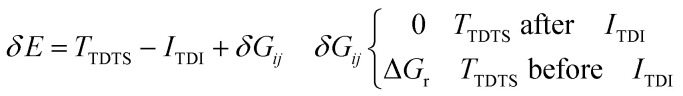
where *T*_TDTS_ and *I*_TDI_ represent the Gibbs free energies of the TDTS (TOF-determining transition state) and the TDI (TOF-determining intermediate), respectively, while *δG*_*ij*_ represents the total Gibbs free energy change of the reaction.

The degree of TOF control (X_TOF_) is a quantitative parameter used to evaluate the extent to which each reaction intermediate or transition state influences the overall reaction rate. X_TOF_ was calculated using the following equation:5
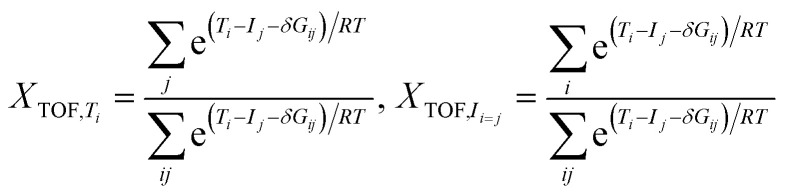


## 3. Results and discussion

### 3.1 Analysis of d–d orbital coupling mechanism between Rh and Co in RhCo_3_

**Fig. 1:**
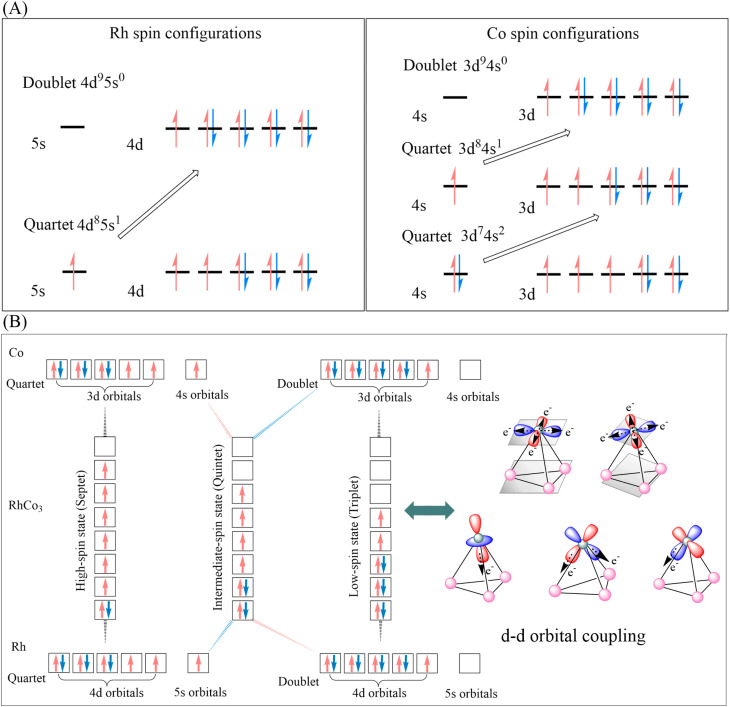
(A) Spin configurations of Rh/Co; (B) d*–*d orbital coupling mechanism of RhCo_3_.

As shown in Fig. 1(A), 5s electrons have a lower energy than 4d electrons, owing to shielding and penetration effects. The electron configuration of Rh is [Kr]4d^8^5s^1^. According to Hund's rule, electrons fill equivalent orbitals with parallel spins, and electrons located within the same orbital have opposite spins (Pauli exclusion principle). Hence, the electrons will preferentially fill the 5s orbital and continue to fill the 4d orbitals (d_*xy*_, d_*yz*_, d_*xz*_, d_*x*^2^−*y*^2^_, d_*z*^2^_) to form the quartet (^4^Rh, ground) state of Rh. Excitation of a single electron from the 5 s to the 4d orbital changes the electronic configuration to 4d^9^5s^0^, resulting in a doublet state (^2^Rh).

The electron configuration of Co is [Ar]3d^7^4s^2^. Owing to the greater penetration effect of the 4s orbital, its energy is lower than that of the 3d orbital, causing electrons to occupy the 4s orbital first, and leading to a low-activity quartet ground state (^4^Co) with three unpaired electrons in the 3d orbital. Low-energy excitation of the 4s electrons of Co results in the formation of a quartet 3d^8^4s^1^ configuration with a single unpaired electron in the 4s orbital, making this configuration more chemically reactive compared to the 3d^7^4s^2^ configuration with two paired electrons in the 4s orbital. Co atoms in this state exhibit higher reactivity. At significantly higher excitation energies, all 4s electrons may move to the 3d orbitals, leading to a 3d^9^4s^0^ electronic configuration, corresponding to a doublet state (^2^Co) with one unpaired electron. This highly excited state, which is challenging to achieve in isolated atoms and unstable, may transiently form in RhCo_3_ complexes.^38^ As shown in Fig. 1(B), the Rh 4d and Co 3d orbitals can generate a strong d–d coupling. The strong spin–orbit coupling (SOC) causes the originally fivefold-degenerate d orbitals of Rh and Co to split into separate energy levels, forming two possible electronic configurations. The first configuration involves a face-to-face electron transfer between the Rh d orbitals and Co, where Rh contributes to form RhCo_3_ through the d_*x*^2^−*y*^2^_ and d_*xy*_ orbitals, corresponding to the higher-energy doubly degenerate orbitals. The other configuration involves the coupling of Rh d_*z*^2^_, d_*xz*_, and d_*yz*_ orbitals with the Co d orbitals *via* vertex-to-vertex electron transfer, forming the lower-energy triply degenerate orbitals. Different filling patterns of d orbitals and combinations of spin orientations result in distinct spin multiplicity states of RhCo_3_.

#### 3.1.1 ^3^[RhCo_3_]

**Fig. 2:**
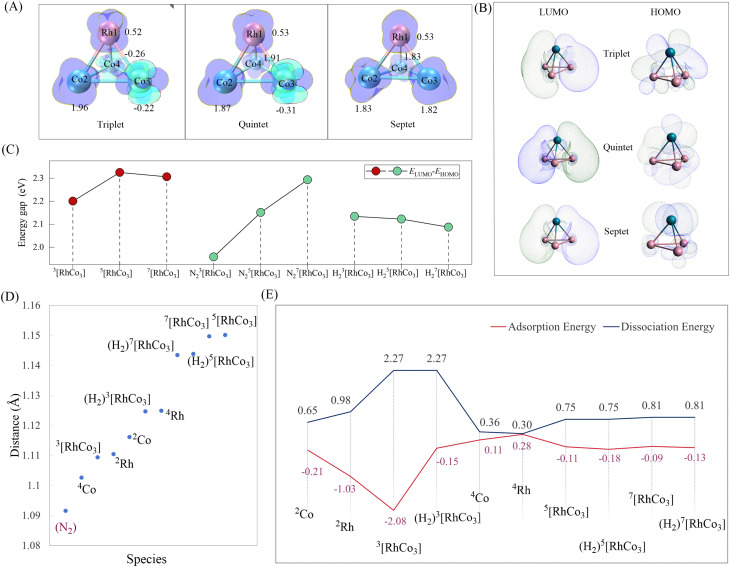
(A) Electron spin density maps of RhCO_3_, the atomic symbols (Rh1, Co2, Co3, Co4) are atomic labels within the molecular structure; (B) illustration of frontier molecular orbitals; (C) energy gap (Δ*E*) diagram of frontier molecular orbitals for various catalysts; (D) N≡N bond lengths of N_2_ adsorbed on various catalysts; (E) N_2_ adsorption and NH_3_ dissociation energies on various catalysts.

As shown in Fig. 2(A), Co exhibits a mixture of high- and low-spin states. Specifically, the Co2 atomic orbitals contain 1.96 *α*-spin electrons, while Co3 and Co4 contain *β*-spin electrons with populations of −0.22 and −0.26, respectively. The Rh atoms possess approximately 0.52 *α*-spin electrons, which can interact with the Co ligands to form weak spin-paired bonds with Co3 and Co4.

#### 3.1.2 ^5^[RhCo_3_]

Co2 and Co4 atoms contribute 1.87 and 1.91 *α* electrons, respectively, while the Co3 atoms possess −0.31 *β* electrons. The asymmetric distribution of *α* and *β* electrons in the quintet RhCo_3_ state has two effects. The high-spin Co2/Co4 atoms facilitate H transfer through the excess *α* electrons, whereas the *β* electrons of Co3 can form weak covalent interactions with the d orbitals of Rh to modulate the stability of reaction intermediates.

#### 3.1.3 ^7^[RhCo_3_]

Rh and Co are coordinated in a quartet state with spin populations of 0.53, 1.83, 1.82, and 1.83 for Rh, Co2, Co3, and Co4, respectively. The d orbitals of Rh and Co contribute 0.72 and 6.28 *α* electrons, with the spin populations of s and p orbitals being −0.60 and −0.41. The total spin multiplicity is predominantly influenced by the strong spin polarization of the d orbitals. The disappearance of the *β* electrons suggests that the *α* electrons of ^7^[RhCo_3_] are strongly delocalized. Although the single-electron densities at the Rh center in the triplet, quintet, and septet states of RhCo_3_ are similar, the unpaired electron of the Co atom exhibits a distinct behavior, highlighting the different properties of the RhCo_3_ bimetallic catalyst across different metal sites and spin states, particularly evident from the varying electron density at the Co atoms.

The frontier molecular orbitals (FMOs) of RhCo_3_ are formed through the contribution of d electrons from Rh and Co (Fig. 2(B)). DFT calculations reveal that the adsorbed N_2_/H_2_ molecules induce additional splitting of Rh 4d and Co 3d orbitals in RhCo_3_, resulting in non-degenerate highest occupied molecular orbital (HOMO) and lowest unoccupied molecular orbital (LUMO) levels. According to FMO theory, a smaller energy gap between frontier orbitals (HOMO–LUMO gap) results in a higher reactivity. The energy gaps between the FMOs after adsorbing N_2_ and H_2_ are shown in Fig. 2(C). The ^3^[RhCo_3_] cluster exhibits the smallest energy gap (2.20 eV → 1.96 eV) upon N_2_ adsorption, while ^7^[RhCo_3_] shows the narrowest gap (2.31 eV → 2.09 eV) after H_2_ adsorption. The reduced post-adsorption gaps observed in both cases indicate enhanced catalytic activities, with ^3^[RhCo_3_] and ^7^[RhCo_3_] showing the highest N_2_ and H_2_ activation abilities, respectively.

### 3.2 Adsorption properties of RhCo_3_ and analysis of electrostatic potential interactions

According to the Sabatier principle,^39–41^ an effective catalyst should exhibit an optimal binding strength, balancing the adsorption of reactants and the desorption of products, with the reaction rate peaking at a moderate adsorption energy. The bond lengths of chemically adsorbed N and H species on the surfaces of Co, Rh, and RhCo_3_ are compared in Fig. 2(D). Upon adsorption, electrons from the d orbitals of the Co, Rh, and RhCo_3_ metals migrate to the antibonding orbitals of nitrogen, leading to an increase in the N≡N bond length. The ^5^[RhCo_3_] and ^7^[RhCo_3_] catalysts exhibit the highest activity, resulting in an increase in the N≡N bond length from 1.09 to 1.15 Å upon N_2_ adsorption, whereas that of (H_2_)^5^[RhCo_3_] and (H_2_)^7^[RhCo_3_] increases to 1.14 Å. RhCo_3_ exhibits distinct active site characteristics near Rh and Co sites, as evidenced by their different catalytic behaviors. The d orbitals of Rh are partially filled and remain unsaturated within the complex, facilitating the spontaneous adsorption of N_2_ and H_2_ on the active Rh sites of RhCo_3_. ^3^[RhCo_3_] displays the lowest N_2_ adsorption energy (−2.08 eV), but at the same time makes the NH_3_ desorption unfavorable (dissociation energy 2.27 eV) (Fig. 2(E)). ^7^[RhCo]_3_ exhibits thermodynamic benefits (SI Tables S1–S3) in addition to a moderate N_2_ adsorption energy (−0.09 eV); following H_2_ adsorption, the N_2_ adsorption energy on (H_2_)^7^[RhCo]_3_ decreases to −0.13 eV, indicating a good resistance to hydrogen poisoning. The NH_3_ desorption is a crucial step in the ammonia synthesis; the calculated NH_3_ desorption barrier from ^7^[RhCo_3_] (0.81 eV) is lower than that of catalysts such as FeMo (1.00 eV) and Fe_3_Co (0.90 eV), and close to that of the highly active RuMo (0.77 eV).^42^ This indicates that ^7^[RhCo_3_] exhibits favorable performance in facilitating NH_3_ desorption.

**Fig. 3:**
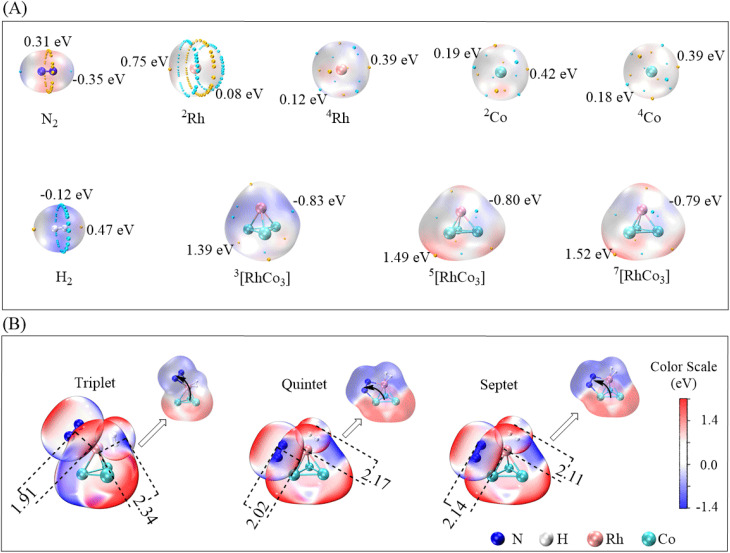
(A) Illustration of electrostatic potential extrema; (B) electrostatic potential penetration of co-adsorbed N_2_ and H_2_ on RhCo_3_ cluster surface (units: Å).

The extrema of the electrostatic potential for each catalyst are shown in Fig. 3(A). The N site displays a negative potential (*V*_s,min_ = −0.35 eV) on its terminal side, attributed to lone-pair electrons, while the bridging side shows a *V*_s,max_ value of 0.31 eV. The minimum potential energy for H_2_ bridging adsorption (*V*_s,min_) is −0.12 eV. The RhCo_3_ cluster shows an increased separation between the positive and negative electrostatic potential regions, owing to the electron cloud density shifting toward the more electronegative Rh. This separation becomes more pronounced with increasing single-electron density. The ^7^[RhCo_3_] cluster exhibits a higher spin polarization (with more unpaired electrons), generating an ESP gradient of 2.31 eV between its positive and negative extrema, which significantly increases the reactivity of its polarized regions, as shown in Fig. 3(B). Both N_2_ and H_2_ adsorb onto the Rh active center in a bridging configuration. The size of the overlapping electrostatic potential regions between RhCo_3_ and N_2_ in the triplet, quintet, and septet states exhibits a gradual increase (1.91 → 2.02 → 2.14 Å), while those of the regions between H_2_ and RhCo_3_ show a gradual decrease (2.34 → 2.17 → 2.11 Å). This trend implies that a higher single-electron spin density results in stronger N_2_ and weaker H_2_ adsorptions. The electronegativity of N causes a shift of the electron density of the Rh–Co cluster toward the outer side of the Rh center in the N direction, leading to its intensification near the –NN moiety. The elongation of the N–N bond and the increased electronegativity of N suggest a weakening of the N≡N bond. This shows that the multiple-spin single electrons of RhCo_3_ can enhance the adsorption ability of Rh within the metal cluster, activating the N–N bond.

### 3.3 Molecular structure optimization and reaction PES calculation

**Fig. 4:**
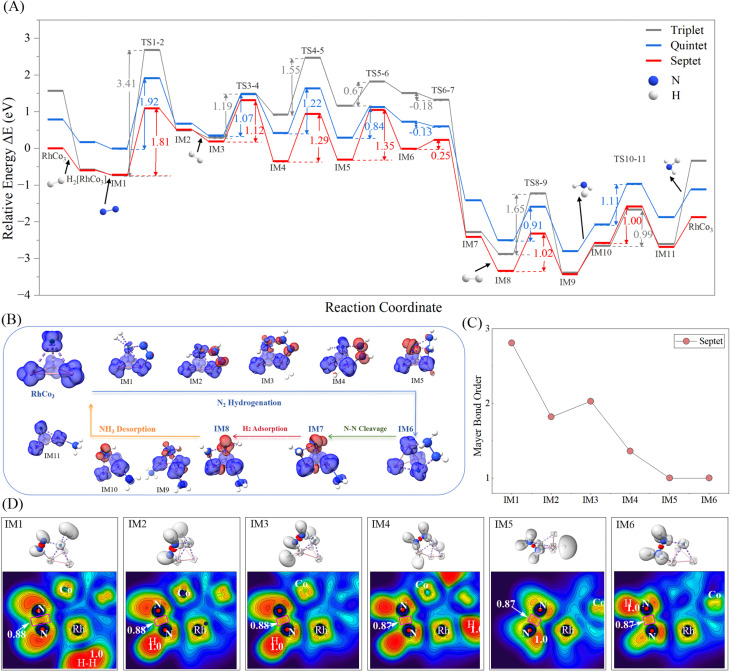
(A) PESs of N_2_ and H_2_ reactions catalyzed by triplet/quintet/septet RhCo_3_; (B) reaction mechanism of N_2_ activation by ^7^[RhCo_3_] and illustration of spin density changes; (C) N–N bond order of partial intermediate species formed on septet RhCo_3_; (D) electronic localization maps of partial intermediates formed on septet RhCo_3_.

The competitive adsorption of H_2_ is thermodynamically favored over that of N_2_, leading to the sequential adsorption of H_2_ and N_2_ at the Rh site. The reaction potential energy surfaces (PESs) for the triplet, quintet, and septet states are shown in Fig. 4(A), and the corresponding energies and geometries are provided in the SI (Fig. S1–S3).

In the uncatalyzed pathway, the transfer of the first H atom is the rate-determining step for the N_2_ reaction with H_2_, with an energy barrier of 5.43 eV.^43^ This is the step with the highest energy barrier in the sequential insertion process of three H_2_ molecules. The catalytic H transfer (TS1-2) reaction on isolated RhCo_3_ exhibits the highest energy barrier (3.41 eV) on the triplet PES, with lower barriers of 1.92 and 1.81 eV observed on the quintet and septet surfaces, respectively. The TS1-2 vibrational frequencies corresponding to different spin states exhibit the typical characteristics of energy barriers for H transfer reactions (*i.e.*, high and thin barriers). As the number of unpaired spin electrons increases, the repulsive interaction between these electrons causes an increase in the Rh–N bond distance, strengthening the exchange interaction between the electrons. This leads to a decrease in the force constants, with the vibrational frequencies corresponding to the H transfer decreasing in the order triplet (−918.61 cm^−1^) > quintet (−839.38 cm^−1^) > septet (−783.93 cm^−1^) (Tables S4–S6). The N hydrogenation reaction generally follows either the alternating hydrogenation or the distal associative hydrogenation pathway. The second H adsorption is the key step in the hydrogenation mechanism. Owing to the presence of the pre-adsorbed H_2_, the Rh site in IM2 exhibits significant steric hindrance. The Co2 site provides additional adsorption space, and the induced positive charge on the NNH moiety (IM2 0.06 eV, Table 1) facilitates the H transfer from Co2 to N, thereby determining the alternating hydrogenation reaction pathway. In this process, the PESs of the three spin states for the H transfer process exhibit similar shapes. The two N atoms undergo successive hydrogenation steps, leading to gradual elongation of the N–N bond and cleavage of the two π bonds between the N atoms. After four H transfers, the RhH_2_N–NH_2_Co2 (IM6) species is formed. The transition from IM6 to IM7 involves the cleavage of the N–N *σ* bond. This is a barrierless reaction in the triplet state (*E*_TS6–7_ < *E*_IM6_), whereas it requires overcoming a barrier of 0.25 eV in the septet state. After the N–N bond cleavage, the system energy significantly decreases, with the NH_3_ dissociation occurring at the Co3 and Co2 sites. The final products are RhCo_3_ and NH_3_, and the overall catalytic cycle is exothermic (Δ*E* = −1.9 eV).

**Table 1:** Atomic charge distribution of intermediates on septet PES

Atomic charge	Rh1	Co2	Co3	Co4
RhCo_3_	0.37	−0.13	−0.12	−0.12
IM1	0.64	−0.10	−0.02	−0.06
IM2	0.79	0.06	−0.10	−0.12
IM3	0.85	−0.21	−0.05	−0.14
IM4	1.24	−0.08	−0.03	−0.11
IM5	0.87	−0.07	−0.23	−0.11
IM6	0.24	−0.09	−0.20	−0.22
IM7	0.77	−0.44	0.16	0.03
IM8	1.12	−0.43	0.20	0.06
IM9	0.98	−0.31	−0.42	0.09
IM10	0.76	−0.29	0.02	0.08
IM11	0.49	−0.55	−0.09	−0.09

The DFT-calculated PESs corresponding to different spin states reveal that the triplet exhibits a more stable adsorption intermediate structure. This stability is due to Rh and Co forming a strong ligand field environment with low spin states in the triplet configuration, with Rh serving as the electron donor (Table 1) and carrying a positive charge, while Co bears a partial negative charge. During H transfer and NH_3_ dissociation, the Rh–N and Co–NH_3_ bonds exhibit shorter lengths due to their high stability under strong ligand field effects, making them highly thermodynamically stable, but kinetically unfavorable. As a result, transition states on the triplet-state PES generally feature higher energy barriers, requiring more energy for NH_3_ dissociation. In the septet state, both Rh and Co adopt a high-spin coordination, promoting the formation of adsorption intermediates with comparatively lower energies. Fig. 4(B) illustrates the changes in the electron spin density; the adsorption of H_2_ induces electron accumulation (red electron cloud) on the Rh surface (IM2) and transfer to the N antibonding orbital, which reduces the N–N bond order and weakens the N≡N bond. This, in turn, leads to the progressive weakening of the π and *σ* bonds of N_2_. The reaction mechanism can be divided into four distinct stages: N_2_ hydrogenation → N–N bond cleavage → H_2_ adsorption → NH_3_ dissociation. As shown in Fig. S4, the variations in metal–metal bond lengths along the reaction coordinate indicate that the RhCo_3_ cluster remains generally stable throughout the catalytic process. The Rh–Co bond lengths exhibit only slight fluctuations, suggesting that the metallic framework of the catalyst possesses high structural strength. A temporary distortion of the Co2–Co4 bond occurs for the IM4 intermediate, likely originating from local structural rearrangements associated with H migration and N_2_ activation. However, the system subsequently recovers its original configuration, demonstrating the excellent structural flexibility and catalytic stability of the RhCo_3_ catalyst.

As the H transfer proceeds, the N–N bond order gradually decreases from 2.81 (co-adsorption state) to 1.82, 2.03, 1.36, and eventually to 1, until complete bond cleavage occurs for the IM7 intermediate, as clearly shown in Fig. 4(C) for the catalytic reaction in the septet state. The electron localization analysis shown in Fig. 4(D) reveals localized electron clouds (red) with covalent bond characteristics between the two N atoms of the N_2_ molecule; moreover, localized electron clouds (gray) are also present between H atoms during the H_2_ adsorption on IM1 and IM2. The ELF value of the N–N bond stabilizes at 0.88, while the electron-localized region of the N–H bond progressively expands as the reaction proceeds. Concurrently, the red-colored covalent character region of the N–N bond gradually shrinks, corresponding to the sequential reduction in bond order during the H-atom transfer.

### 3.4 Bimetallic synergistic effect of Rh and Co

According to the charge distribution in the septet reaction system, Rh primarily acts as the electron-donating center, with a positive electron cloud density (Table 1).

In RhCo_3_, Rh carries a positive charge of 0.37, which results in a strong adsorption of H_2_ molecules. In the co-adsorbed IM1, the electron densities of Rh1, Co2, Co3, and Co4 are all reduced, and the Co atoms may receive electrons back-donated from the N atom *via* the Rh center, thereby influencing the electron cloud densities of both Rh and Co atoms. Mulliken charge analysis reveals that the Co2 atom in the IM2 intermediate carries a net positive charge of +0.06, demonstrating its H_2_ adsorption ability. As the positive charge on Rh increases and its electron cloud density decreases (in IM1, IM2, IM4, and IM8), the electron cloud density of Co exhibits a simultaneously decrease. In IM9 and IM11, after the formation of the NH_3_ molecule, Co donates electrons back to N, leading to an increase in the electron cloud density of Co. The results indicate that the Co and Rh atoms can synergistically influence the catalytic reactions by dynamically adjusting the electron cloud around the Rh atoms to meet the reaction requirements. Table 2 shows that the total spin population of all intermediates in the septet state remains around 6, with the spin population of Rh ranging from −0.76 to 0.53. In some of the intermediates (IM5, IM7, IM8, IM10), the transfer of d electrons from Rh to N leads to a negative spin population for the Rh center, while that of the Co2 atoms significantly increases to above 2.4 (2.56, 2.48, 2.51, 2.58) to maintain the overall spin stability. During the H-to-N transfer process, the Rh atom in RhCo_3_ predominantly acts as the reactant activation site by engaging with the reactant molecules and activating the N≡N bond. The Co atom supports this role by dynamically adjusting the electron cloud environment around Rh through electron transfer and spin modulation, and potentially aiding in maintaining the structural stability of the active center of the catalyst.

**Table 2:** Spin population of intermediates on septet PES

Spin population	Rh1	Co2	Co3	Co4
RhCo3	0.53	1.83	1.82	1.83
IM1	0.03	1.99	2.04	2.04
IM2	0.34	1.96	2.04	2.10
IM3	0.07	1.79	2.10	2.32
IM4	0.32	2.38	1.91	2.17
IM5	−0.65	2.56	2.13	2.02
IM6	0.54	1.77	1.87	1.82
IM7	−0.76	2.48	2.22	2.00
IM8	−0.59	2.51	2.10	1.91
IM9	−0.22	2.33	2.16	1.78
IM10	−0.61	2.58	1.98	1.99
IM11	0.37	2.01	1.84	1.84

### 3.5 Analysis of reaction pathways and catalytic TOF

**Fig. 5:**
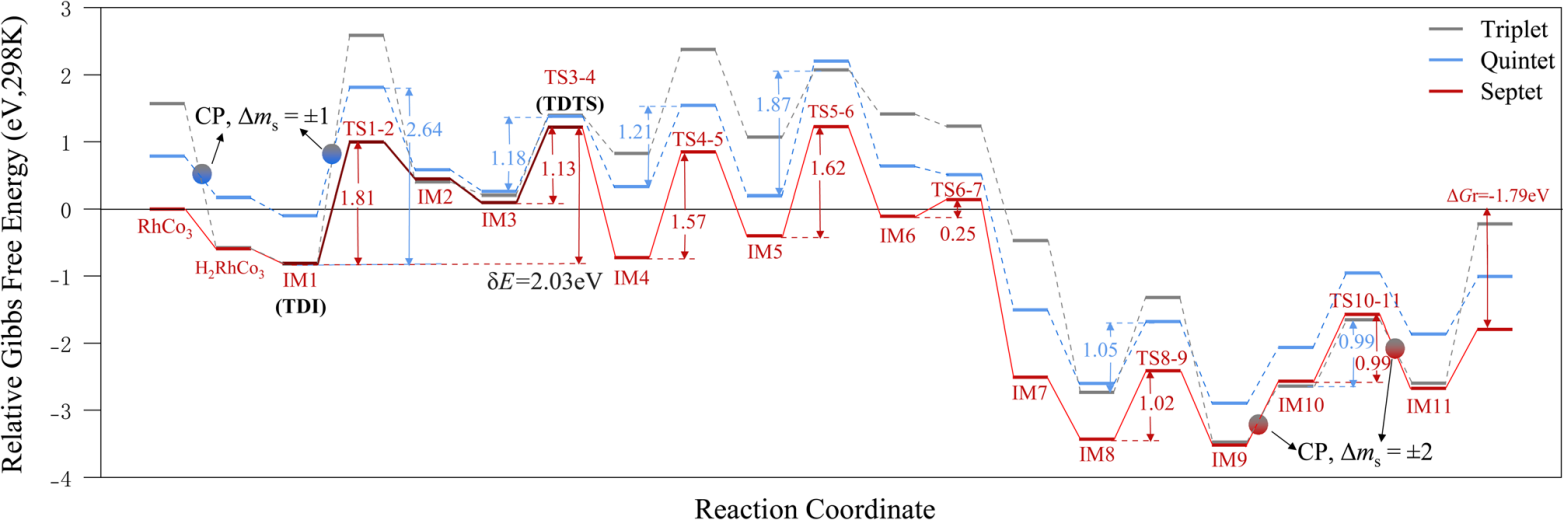
Relative Gibbs free energy diagrams for RhCo_3_-catalyzed N_2_ + 3H_2_ →2NH_3_ reaction at 298 K.

Fig. 5 shows the relative Gibbs free energy diagrams (298 K) for the RhCo_3_-catalyzed N_2_ + 3H_2_ → 2NH_3_ reaction across the triplet, quintet, and septet states. The total spin quantum number difference Δ*m*_s_ = ±1 for the triplet and quintet states allows potential energy surface crossings, with multiple crossing points (CPs) distributed before the H_2_ adsorption and H transfer barriers. However, the energy gaps of the adsorbates and transition states vary significantly (ranging from 0.99 to 2.64 eV), requiring the overcoming of high energy barriers, and the energies of all states remain higher than those observed on the septet PES. For the triplet–septet transition, Δ*m*_s_ = ±2, which is forbidden, implying minimal PES mixing at crossing points. The Gibbs free energy barriers for consecutive hydrogenations over the septet state ^7^[RhCo_3_] are 1.81, 1.13, and 1.02 eV. Compared with the Nb-based bimetallic system Nb_2_Rh (1.70, 1.74, and 1.80 eV), ^7^[RhCo_3_] exhibits lower energy barriers for the sequential hydrogenation steps, showing a notable kinetic advantage in the second and third hydrogenation processes.^44^

**Fig. 6:**
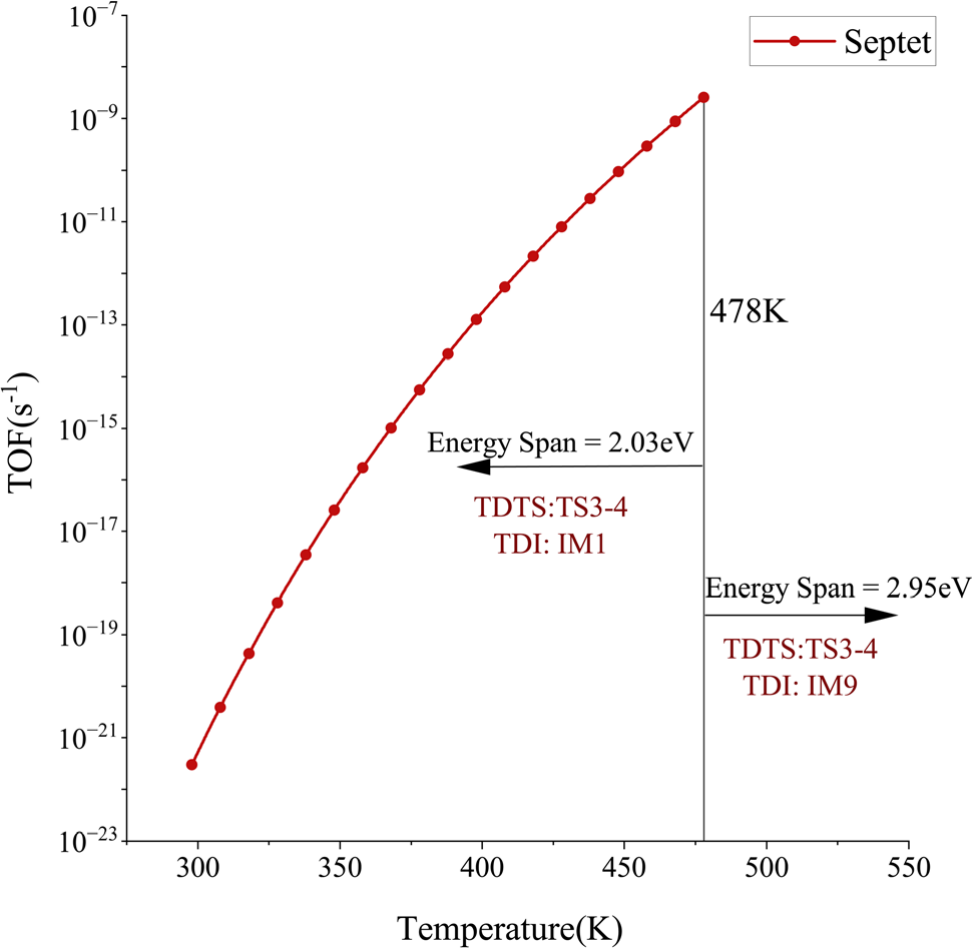
The relationship between the TOF and temperature for the ^7^[RhCo_3_]-catalyzed N_2_ reaction for ammonia synthesis.

Theoretical calculations with the energy span model were performed to determine the TOF values and rate-determining states of the RhCo_3_-catalyzed N_2_ and H_2_ cyclic reactions. The calculations reveal that the N_2_ activation reaction over septet RhCo_3_ exhibits high thermal sensitivity within the temperature range of 298–478 K. The turnover-determining intermediate (TDI) is identified as IM1, while the turnover-determining transition state (TDTS) corresponds to TS3-4, defining the rate-determining region between these two states. The adsorption of N_2_ and the subsequent cleavage of the N–N *σ* bond are the key steps influencing the overall reaction rate. The energy span (δ*E*) is calculated to be 2.03 eV, and the TOF of RhCo_3_ increases exponentially from 2.98 × 10^−22^ to 2.53 × 10^−9^ s^−1^, following a typical Arrhenius behavior (Fig. 6). When the temperature exceeds 478 K, the relative free energy values of the intermediates changes: the TDTS remains positioned at TS3-4, while the TDI shifts to IM9, which is located beyond the TDTS. Hence, the energy span increases to δ*E* = *T*_TDTS_ − *I*_TDI_ + *δG*_*ij*_ = 2.95 eV, which may lead to a decline in the TOF. This result suggests that, at higher temperatures, the influence of the N–N bond dissociation on the RhCo_3_ activity is reduced, and the desorption step becomes the dominant process.

## 4. Conclusions

In this study, we have investigated the mechanism of the N_2_ + 3H_2_ → 2NH_3_ reaction promoted by the d–d coupling-based bimetallic catalyst RhCo_3_, focusing on the synergistic and spin effects between Rh and Co. The results show that the unsaturated d orbitals of Rh and Co atoms in RhCo_3_, combined with the significant electronegativity difference, induce a marked d–d orbital coupling. This interaction generates a favorable bimetallic synergy and induces spin effects, which collectively modulate the N_2_ activation ability. Septet RhCo_3_ exhibits an H_2_ adsorption energy of −0.59 eV, a subsequent N_2_ adsorption energy of −0.13 eV, as well as NH_3_ dissociation energies of 0.85 and 0.81 eV, indicating moderate adsorption and dissociation energies with low energy barriers for H transfer. In the H transfer process to N, the Rh atom acts as electron regulation center and reactant activation site, while the Co metal contributes to enhance the electron back-donation effect. This synergistic effect is primarily attributed to the establishment of a dynamic electron-transfer mechanism between Rh and Co within the polymetallic centers of the RhCo_3_ cluster. Between 298 and 478 K, the septet RhCo_3_ catalyst exhibits a significant increase in TOF, peaking at 478 K. The d–d coupling-based bimetallic catalyst RhCo_3_ effectively promotes N_2_ activation through bimetallic synergy and spin effects. This catalyst achieves an optimal balance between the adsorption energies of reactants and key intermediates, the product dissociation energies, and the H transfer barriers, enabling an efficient NH_3_ synthesis. The spin effects of Rh and Co play a critical role in the catalytic process. In conclusion, this study provides theoretical support for the design of multimetallic catalysts with properties tunable *via* spin effects.

## Author contributions

Jingjing Wu carried out the investigation and wrote the manuscript. Haixiong Shi contributed to supporting roles in the literature survey. Yongcheng Wang developed the methodology.

## Conflicts of interest

There are no conflicts to declare.

## Supplementary Material

RA-015-D5RA06945A-s001

## Data Availability

The data supporting the findings of this study are available within the article and its supplementary information (SI). Supplementary information: optimized geometries (Cartesian coordinates), energy values, and transition-state vibrational frequencies. See DOI: https://doi.org/10.1039/d5ra06945a.
