# Supplementary material for: Mechanistic insights into N2 activation by RhCo3via d–d orbital coupling
Source: RSC Adv. 2025 Nov 6;15(50):43187–97. doi: 10.1039/d5ra06945a (PMC12590544; doi:10.1039/d5ra06945a)
Supplement: RA-015-D5RA06945A-s001 [file RA-015-D5RA06945A-s001.pdf]

Supplementary Information (SI) for RSC Advances.
This journal is © The Royal Society of Chemistry 2025

ARTICLE

Supplementary Information：

Mechanistic Insights into N_2 Activation by $RhCo_3$ via d-d Orbital Coupling

Jing Jing Wu *[a], Hai Xiong Shi [a] and Yong-Cheng Wang [b]

[a]*(School of Chemical Engineering, Lanzhou University of Arts and Science , Lanzhou 730010, China)*

[b]*(College of Chemistry and Chemical Engineering, Northwest Normal University, LanZhou 730070, China)*

Table S1 Energies *E* (a.u.), relative energies Δ*E* (eV, relative to $^7[RhCo_3]$), Gibbs free energies *G* (a.u.), and relative Gibbs free energies Δ*G* (kcal/mol, relative to $^7[RhCo_3]$) of various intermediates and transition states in the ammonia synthesis reaction via N_2 activation and hydrogenation by $^3[RhCo_3]$

Species	*E*(a.u.)	Δ*E*(eV)	*G* (a.u.)	Δ*G*(eV)
$RhCo_3(+N_2+3H_2)$	-661.13	1.57	-661.13	1.57
$H_2RhCo_3(+N_2+2H_2)$	-661.21	-0.58	-661.21	-0.58
IM1(+2H_2)	-661.22	-0.73	-661.22	-0.82
TS1-2(+2H_2)	-661.09	2.68	-661.09	2.59
IM2(+2H_2)	-661.17	0.49	-661.17	0.40
IM3(+H_2)	-661.18	0.30	-661.18	0.20
TS3-4(+H_2)	-661.14	1.49	-661.14	1.40
IM4(+H_2)	-661.16	0.92	-661.16	0.82
TS4-5(+H_2)	-661.10	2.47	-661.10	2.37
IM5(+H_2)	-661.15	1.16	-661.15	1.07
TS5-6(+H_2)	-661.13	1.83	-661.11	2.07
IM6(+H_2)	-661.14	1.51	-661.14	1.42
TS6-7(+H_2)	-661.14	1.33	-661.14	1.23
IM7(+H_2)	-661.28	-2.28	-661.21	-0.47
IM8	-661.30	-2.88	-661.29	-2.73
TS8-9	-661.24	-1.23	-661.24	-1.32
IM9	-661.32	-3.39	-661.32	-3.48
IM10	-661.29	-2.65	-661.29	-2.64
TS10-11(+NH_3)	-661.25	-1.66	-661.25	-1.65
IM11(+NH_3)	-661.29	-2.60	-661.28	-2.60
$RuCo_3(+2NH_3)$	-661.20	-0.33	-661.20	-0.22

Table S2 Energies *E* (a.u.), relative energies Δ*E* (eV, relative to $^7[RhCo_3]$), Gibbs free energies *G* (a.u.), and relative Gibbs free energies Δ*G* (kcal/mol, relative to $^7[RhCo_3]$) of various intermediates and transition states in the ammonia synthesis reaction via N_2 activation and hydrogenation by $^5[RhCo_3]$

Species	*E*(a.u.)	Δ*E*(eV)	*G* (a.u.)	Δ*G*(eV)
$RhCo_3(+N_2+3H_2)$	-661.16	0.79	-661.16	0.79
$H_2RhCo_3(+N_2+2H_2)$	-661.19	0.17	-661.18	0.17
IM1(+2H_2)	-661.19	-0.01	-661.19	-0.10

TS1-2(+2H_2)	-661.12	1.91	-661.12	1.82
IM2(+2H_2)	-661.17	0.68	-661.17	0.58
IM3(+H_2)	-661.18	0.35	-661.18	0.26
TS3-4(+H_2)	-661.14	1.48	-661.14	1.38
IM4(+H_2)	-661.18	0.42	-661.18	0.33
TS4-5(+H_2)	-661.13	1.64	-661.13	1.54
IM5(+H_2)	-661.18	0.29	-661.18	0.20
TS5-6(+H_2)	-661.15	1.13	-661.11	2.20
IM6(+H_2)	-661.17	0.73	-661.17	0.64
TS6-7(+H_2)	-661.17	0.60	-661.17	0.51
IM7(+H_2)	-661.24	-1.41	-661.24	-1.51
IM8	-661.28	-2.50	-661.28	-2.60
TS8-9	-661.25	-1.59	-661.25	-1.68
IM9	-661.30	-2.80	-661.30	-2.90
IM10	-661.27	-2.07	-661.26	-2.06
TS10-11(+NH_3)	-661.23	-0.96	-661.22	-0.95
IM11(+NH_3)	-661.26	-1.87	-661.26	-1.86
$RuCo_3$(+2NH_3)	-661.23	-1.12	-661.23	-1.01

Table S3 Energies *E* (a.u.), relative energies Δ*E* (eV), Gibbs free energies *G* (a.u.), and relative Gibbs free energies Δ*G* (kcal/mol) of various intermediates and transition states in the ammonia synthesis reaction via N_2 activation and hydrogenation by ^{7}[$RhCo_3$]

**Species**	***E*(a.u.)**	**Δ*E*(eV)**	***G* (a.u.)**	**Δ*G*(eV)**
$RhCo_3$(+N_2+3H_2)	-661.19	0.00	-661.19	0.00
H_2RhCo_3(+N_2+2H_2)	-661.21	-0.59	-661.21	-0.59
IM1(+2H_2)	-661.22	-0.72	-661.22	-0.81
TS1-2(+2H_2)	-661.15	1.09	-661.15	0.99
IM2(+2H_2)	-661.17	0.51	-661.17	0.44
IM3(+H_2)	-661.19	0.19	-661.19	0.10
TS3-4(+H_2)	-661.14	1.31	-661.14	1.22
IM4(+H_2)	-661.21	-0.35	-661.22	-0.72
TS4-5(+H_2)	-661.16	0.94	-661.16	0.85
IM5(+H_2)	-661.20	-0.30	-661.20	-0.40
TS5-6(+H_2)	-661.15	1.05	-661.14	1.22
IM6(+H_2)	-661.19	-0.01	-661.19	-0.11
TS6-7(+H_2)	-661.18	0.24	-661.18	0.14
IM7(+H_2)	-661.28	-2.41	-661.28	-2.51
IM8	-661.31	-3.34	-661.31	-3.43
TS8-9	-661.28	-2.32	-661.28	-2.41
IM9	-661.32	-3.43	-661.32	-3.52
IM10	-661.29	-2.58	-661.28	-2.57
TS10-11(+NH_3)	-661.25	-1.58	-661.25	-1.57
IM11(+NH_3)	-661.29	-2.68	-661.29	-2.67
$RhCo_3$(+2NH_3)	-661.26	-1.87	-661.25	-1.79

Table S4 The first five vibrational frequencies (including the imaginary frequency) of the triplet transition state (cm^{-1})

Species	1	2	3	4	5
TS1-2	-783.9318	52.1696	63.5452	104.4452	131.6870
TS3-4	-1164.1184	55.5642	68.4581	85.6892	116.2556

 This journal is © The Royal Society of Chemistry 20xx

TS4-5	-1162.5208	52.5708	68.7463	87.4477	108.4471
TS5-6	-1127.0987	45.6050	79.8881	107.9228	127.2236
TS6-7	-553.2445	44.5570	61.3309	88.5467	106.3171
TS8-9	-563.1918	41.4197	63.9007	86.2873	102.6238
TS10-11	-868.0769	64.6705	100.3152	121.1623	152.2344

Table S5 The first five vibrational frequencies (including the imaginary frequency) of the quintet transition state (cm^{-1})

Species	1	2	3	4	5
TS1-2	-839.3775	54.1215	74.1799	108.8559	119.7479
TS3-4	-1096.8517	30.7386	66.1646	114.1333	124.0636
TS4-5	-1155.2221	54.1644	74.6342	93.3254	135.3367
TS5-6	-1088.4156	26.7209	89.9297	133.8087	146.2059
TS6-7	-714.9289	55.1293	57.1789	109.6351	116.3615
TS8-9	-622.1250	52.2650	61.0756	82.4063	90.0432
TS10-11	-1099.6943	75.3150	88.9982	126.4127	129.8162

Table S6 The first five vibrational frequencies (including the imaginary frequency) of the septet transition state (cm^{-1})

Species	1	2	3	4	5
TS1-2	-918.6073	55.6382	68.7934	118.5394	122.0825
TS3-4	-1170.2068	41.4306	84.6792	112.0154	127.2964
TS4-5	-1279.9622	29.5490	70.8560	86.5342	113.6046
TS5-6	-1122.6747	26.7999	66.0249	97.6446	118.3692
TS6-7	-699.0756	60.5191	64.2646	113.4229	125.6823
TS8-9	-700.1676	51.3370	63.7354	85.6434	105.7295
TS10-11	-896.5188	23.5349	80.3066	121.2988	153.2536

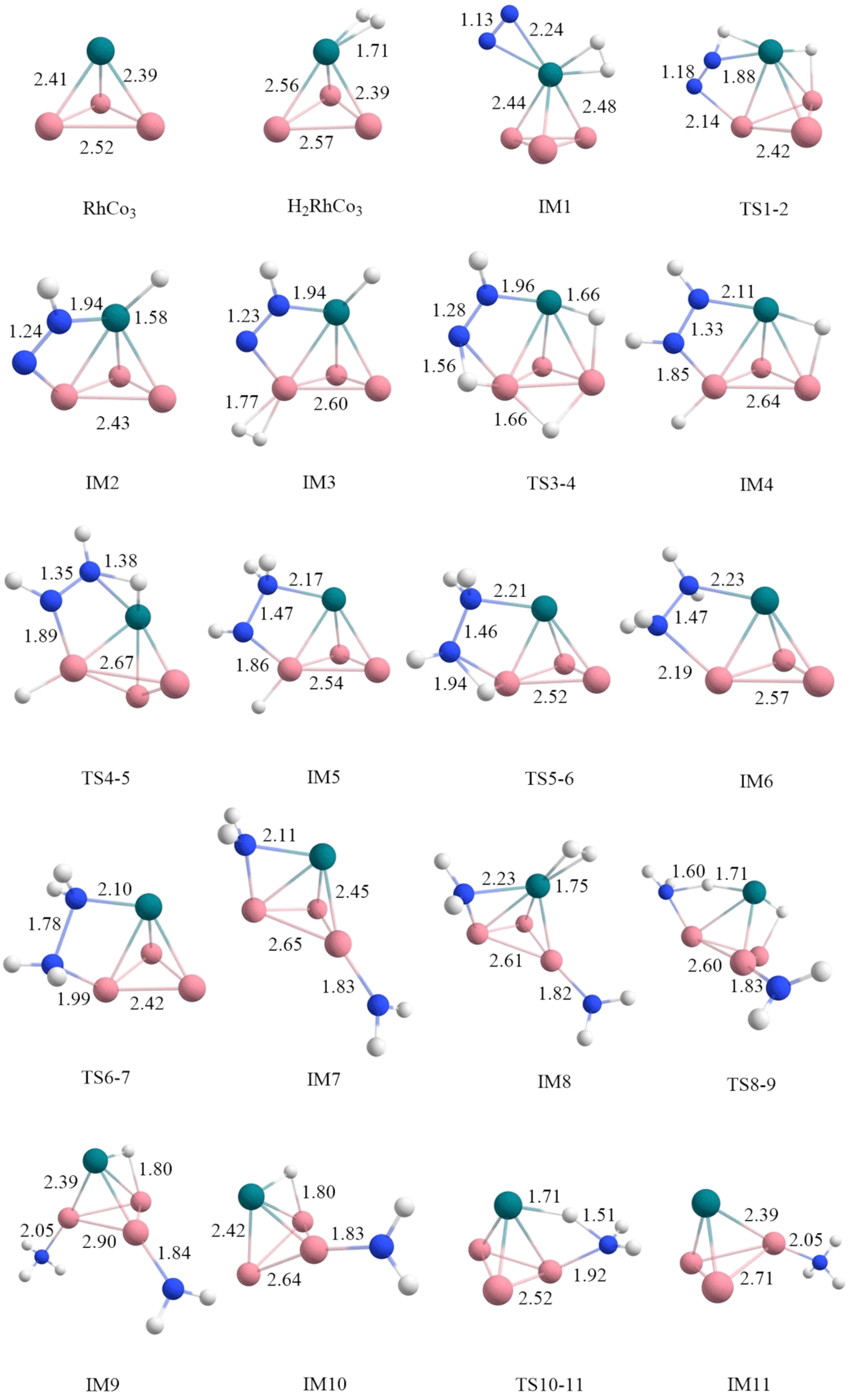

Fig. S1 Configurations of various intermediates and transition states in the ammonia synthesis via N_2 hydrogenation activated by $^3[RhCo_3]$

 This journal is © The Royal Society of Chemistry 20xx

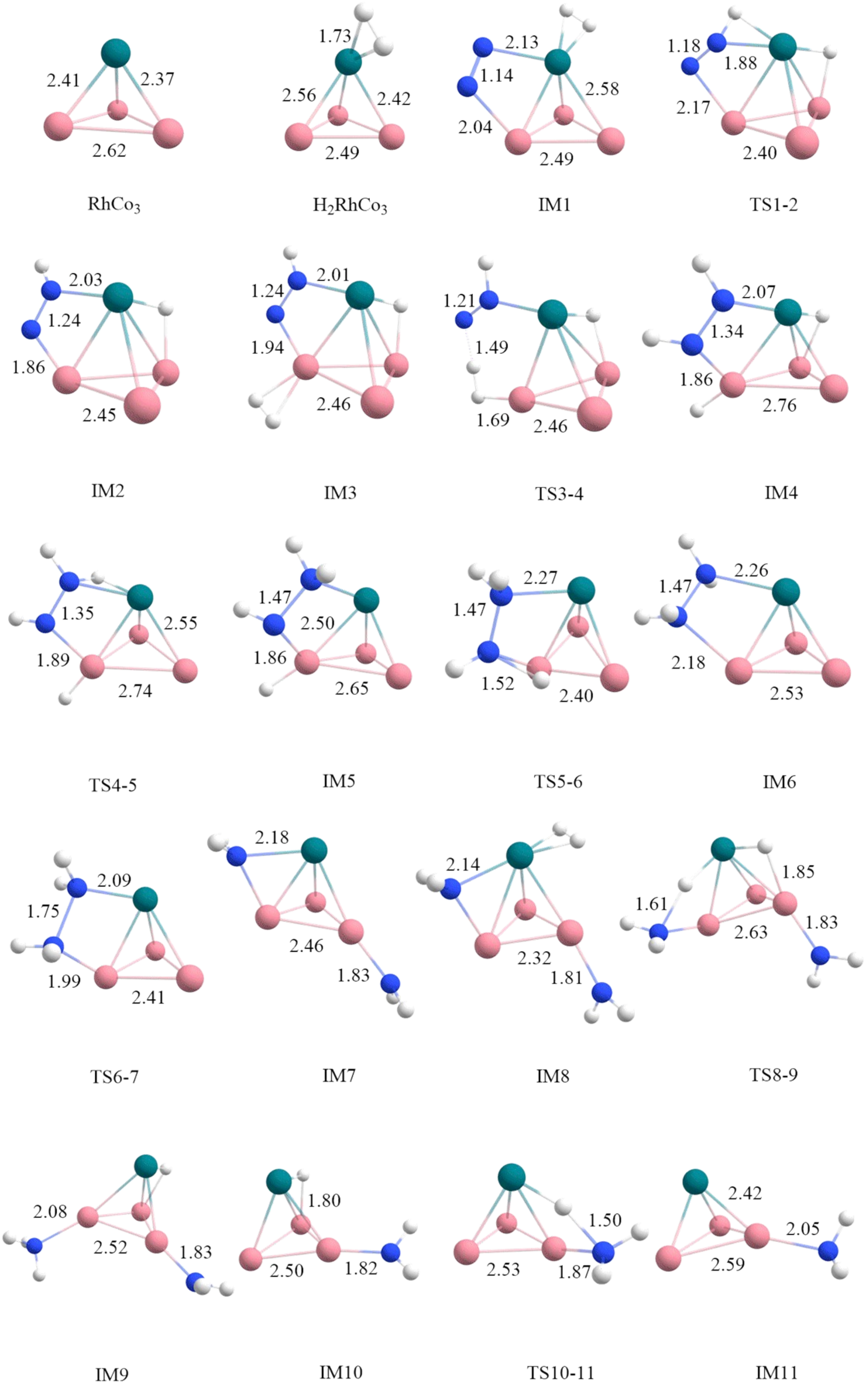

Fig. S2 Configurations of various intermediates and transition states in the ammonia synthesis via N_2 hydrogenation activated by $^5[RhCo_3]$

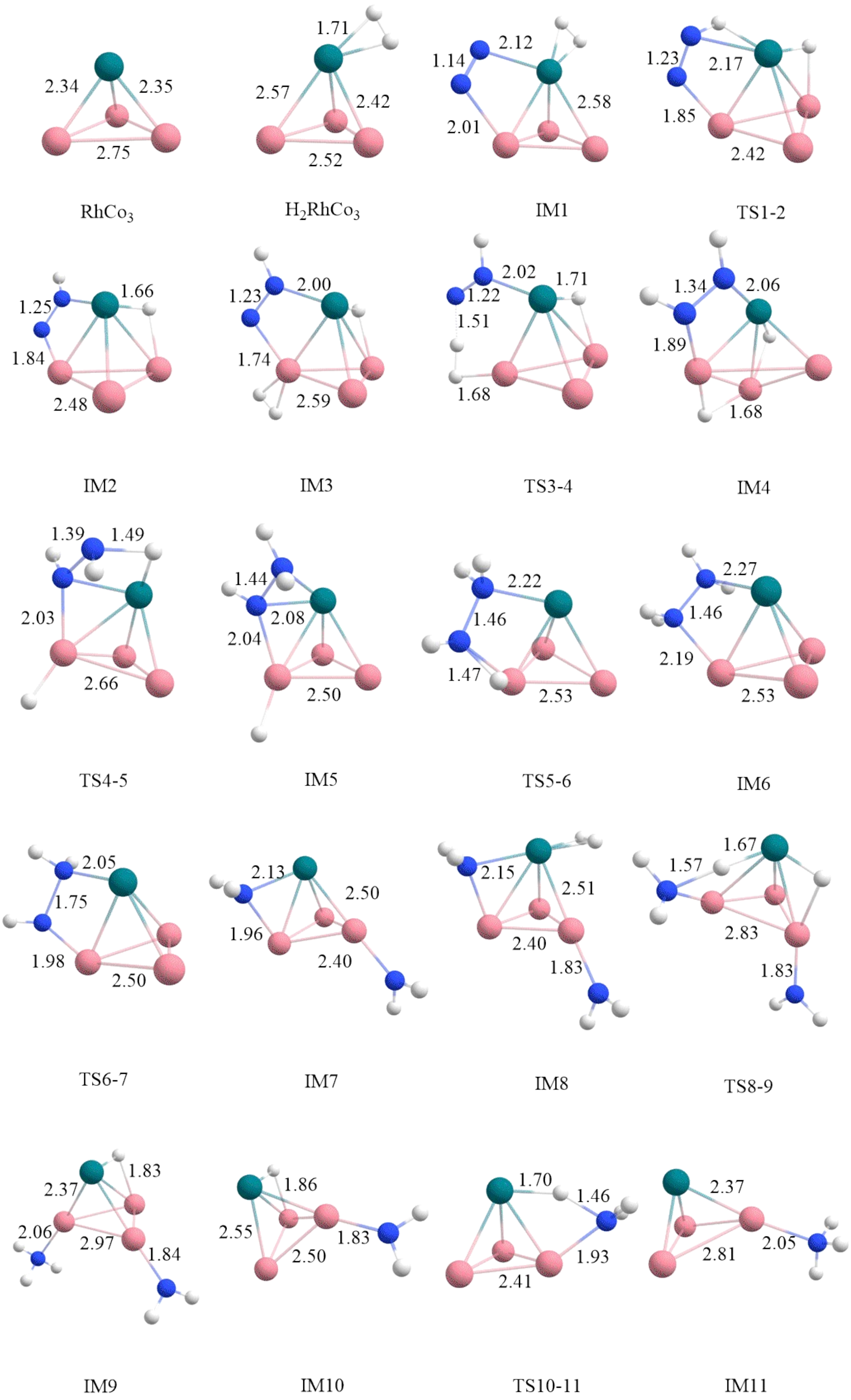

Fig. S3 Configurations of various intermediates and transition states in the ammonia synthesis via N_2 hydrogenation activated by $^7[RhCo_3]$

 This journal is © The Royal Society of Chemistry 20xx

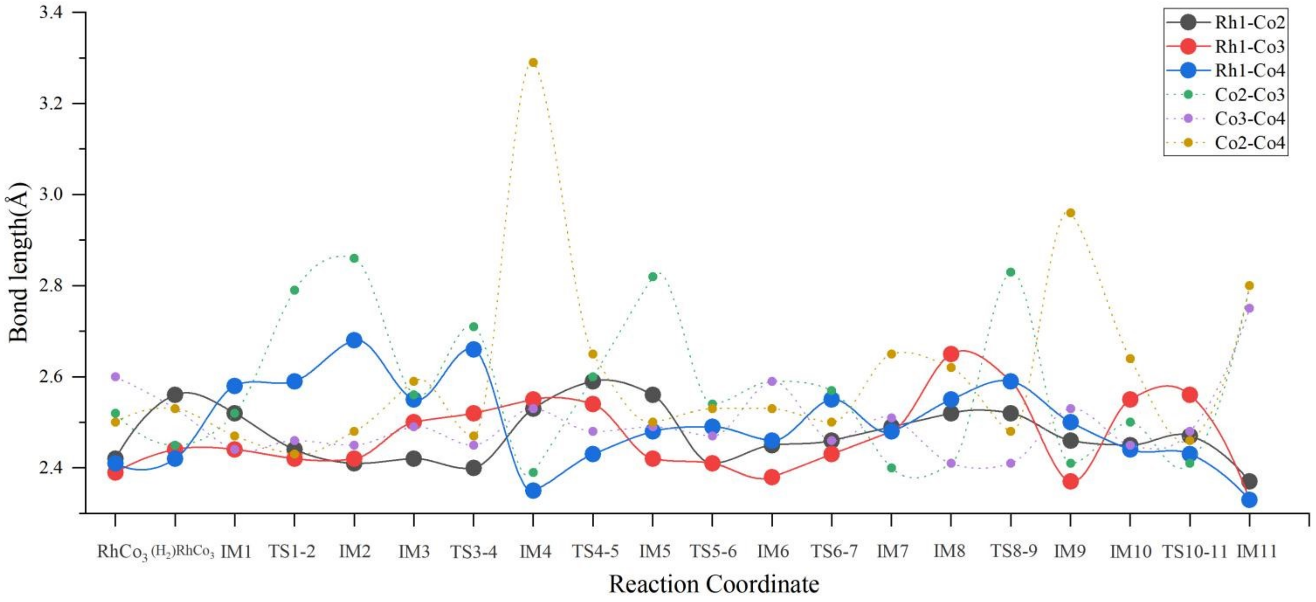

Fig. S4 The variation of Rh-Co and Co-Co distances of on the septet potential energy surface

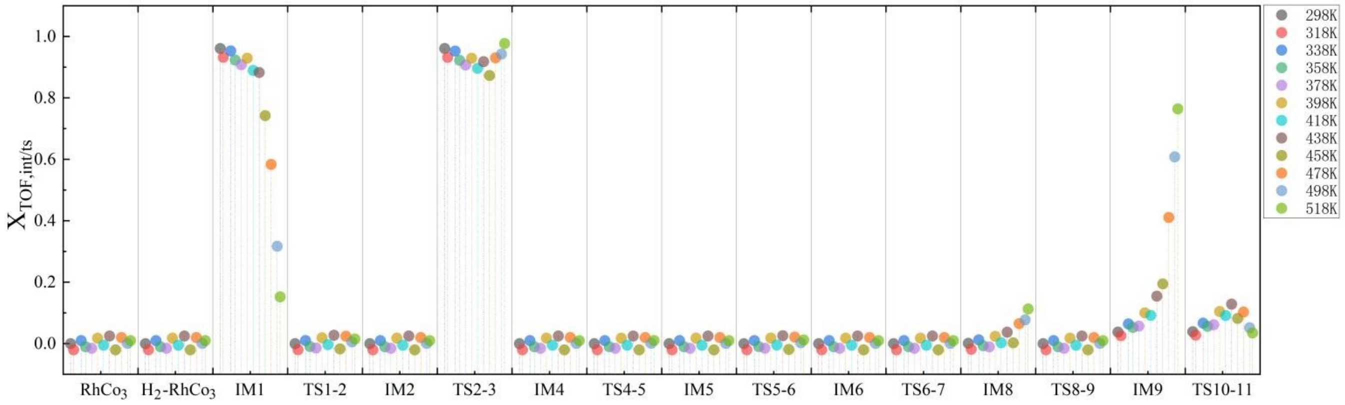

Fig. S5 X_{TOF} of transition states and intermediates on the septet potential energy surface(298~518K)

Optimized Cartesian Coordinates (in Å) for the Intermediate and Transition State Structures

Triplet reaction system

$RhCo_3$

Rh	-1.20141000	-0.12677000	-0.17902400
Co	0.93854700	0.05132000	-1.29930700
Co	0.38398600	1.38845900	0.76103800
Co	0.67981700	-1.22849700	0.83664300

H_2RhCo_3

Rh	-1.25187500	1.28071000	0.60732600

Co	-2.16668300	1.61857700	-1.75764200
Co	0.28387800	1.06023700	-1.21045900
Co	-1.75977300	-0.67513600	-0.67554700
H	-0.08461200	0.90325500	1.80037200
H	-0.71106100	0.30271100	1.89436700

IM1

Rh	0.88741300	-0.51287400	-0.07895400
Co	-0.67082600	1.48896200	-0.54168700
Co	-1.43287500	-0.84282300	-0.81424200
Co	-0.89525800	0.07555300	1.50736900
N	3.05209600	0.06299000	0.01328800
N	2.56049000	1.06378400	-0.13394200
H	0.45192000	-2.16325300	0.00842100
H	1.29824200	-2.13054100	0.30019000

IM2

Rh	0.78944000	-0.85239200	-0.44656800
Co	0.05386100	1.53632600	0.09745100
Co	-1.77339700	0.13596700	-0.82233300
Co	-0.87880300	-0.59465700	1.33536700
N	2.26983300	0.23216000	0.16982100
N	2.01843200	1.42515700	0.29931900
H	1.38035700	-2.25997700	-0.02372700
H	3.23213500	-0.07979000	0.35221500

IM3

Rh	0.80009300	-0.96612800	-0.33237100
Co	0.07040500	1.53700100	0.12479900
Co	-1.68087600	0.06705100	-0.94339800
Co	-0.99579600	-0.59049300	1.28259000
N	2.27825700	0.25051400	0.04072800
N	2.04841100	1.45257200	0.12069500
H	1.57270600	-2.10668200	0.43731000
H	3.24695600	-0.06231600	0.18123700
H	-0.75422400	3.07669600	0.34690400
H	0.01290400	3.28036000	0.33352200

IM4

Rh	0.42231200	-1.22989800	-0.16064800
Co	0.51172000	1.47516200	0.15001400
Co	-1.29231800	0.40873300	-1.18522300
Co	-1.36458000	-0.06550600	1.18749100
N	2.33459800	-0.35838000	0.02897700
N	2.28394800	0.95019500	0.27348700
H	-0.53679700	-1.62678300	1.20251600
H	3.22887100	-0.67841100	-0.33653600
H	0.75736200	2.94809500	0.06674400
H	3.13648600	1.46330300	0.06757100

IM5

Rh	0.34726300	-1.16144300	-0.04840900
Co	0.56030200	1.35386300	-0.03676800
Co	-1.46526700	0.17801900	-1.15454200
Co	-1.25991300	0.16617700	1.28647100

This journal is © The Royal Society of Chemistry 20xx

N	2.39977100	-0.45855200	-0.00218700
N	2.37763200	1.00559800	0.10691900
H	2.87974300	-0.82071900	0.81536300
H	2.90489000	-0.77456900	-0.82594400
H	0.70176000	2.81271400	-0.42505900
H	2.89663100	1.37057700	-0.68840200

IM6

Rh	-0.34675100	-1.06055900	-0.17754100
Co	-0.42291300	1.39028700	-0.13384400
Co	1.07910100	0.10810400	1.41235400
Co	1.65251400	0.13785700	-1.00082400
N	-2.45262600	-0.48483900	0.27067400
N	-2.55659200	0.88628400	-0.23728200
H	-3.31288900	-0.99905300	0.09603100
H	-2.30391600	-0.41766100	1.27358800
H	-2.65929500	0.80839200	-1.24400400
H	-3.39052200	1.34468700	0.13246500

IM7

Rh	0.80360200	-0.83456800	-0.64851700
Co	-1.41279800	-0.41336400	0.31687100
Co	0.73001900	0.85476300	1.23272500
Co	-0.25505500	1.39774900	-1.01768400
N	2.03051500	-0.61029000	1.05244000
N	-3.21318700	-0.64960400	0.53533900
H	2.99460700	-0.44460000	0.78260100
H	2.02043700	-1.39599800	1.69414800
H	-3.92230500	-0.50942200	-0.16926800
H	-3.65461000	-0.93215200	1.39969700

IM8

Rh	0.90543100	-0.58326700	-0.75662400
Co	-1.29672900	0.17180200	0.08802900
Co	0.88985700	1.07646400	1.20639600
Co	0.06459200	2.04116300	-0.81934100
N	1.91089300	-0.56341300	1.23685700
N	-2.79164800	-0.82711000	0.36965400
H	2.91795500	-0.54739500	1.12968100
H	1.67371300	-1.34765200	1.83038000
H	-3.10677900	-1.52499200	-0.29190800
H	-3.03525400	-1.14511400	1.29997300
H	1.09230700	-2.29998900	-1.04867000
H	0.71803300	-2.02716700	-1.75266900

IM9

Rh	-0.06014200	1.39384300	-0.51061300
Co	-1.36116400	-0.70714500	-0.35469400
Co	1.50985200	-0.37059100	-0.17857000
Co	-0.27045500	0.12638800	1.62590600
N	2.92929400	-1.85348100	-0.25501200
N	-2.60560900	-2.04576500	-0.57338200
H	2.79780700	-2.48738500	0.52762000
H	3.87596400	-1.48957600	-0.21080600
H	-3.18748000	-2.34942500	0.19609100

H	-2.41642900	-2.84654100	-1.16340200
H	-0.18096400	1.82513400	1.03557800
H	2.83939700	-2.39401700	-1.11009900

IM10

Rh	0.56091100	1.10191400	-0.32298100
Co	-1.43681600	-0.24601700	-0.20183000
Co	0.91317900	-1.22123600	-0.90355600
Co	0.67871600	-0.42404400	1.52549600
N	-3.20564600	-0.00863100	0.19230000
H	-3.61348100	0.82568100	0.58653600
H	-3.94082000	-0.64575900	-0.08162300
H	0.56572300	1.35936900	1.34014000

IM11

Rh	0.63762800	-1.15995800	0.48899300
Co	-0.92252000	0.55732200	-0.08702400
Co	0.47835100	0.48593000	2.22780400
Co	1.83223700	0.79819800	-0.03303000
N	-2.42074600	1.80666700	-0.72948900
H	-2.25418300	2.76234700	-0.42871000
H	-3.31450600	1.50726900	-0.35209800
H	-2.48796500	1.80032200	-1.74259900

TS1-2

Rh	0.53867600	-1.05515200	0.27840900
Co	0.42991800	1.44333600	-0.45691500
Co	-1.40713000	-0.41868400	-1.06117600
Co	-1.19111400	0.63355700	1.15179600
N	2.13963500	-0.09121300	0.11277400
N	2.50294500	0.93386000	-0.35217300
H	-0.59057400	-1.98769300	-0.69249100
H	2.39691300	-1.20062500	-0.27018800

TS3-4

Rh	-1.05986000	-0.89452300	-0.09659800
Co	0.38141600	1.52172600	0.09339500
Co	1.05499200	-0.56902200	1.29161200
Co	1.28818400	-0.30760700	-1.16587500
N	-2.01423700	0.82659700	-0.03866700
N	-1.39512300	1.87011000	0.37646200
H	-0.35240900	-0.94187900	-1.58069400
H	-2.76636000	0.99004900	-0.71281700
H	-0.54199500	2.53787300	-0.68668300
H	1.65599000	1.37292200	-0.95400400

TS4-5

Rh	0.40856200	-1.05667600	-0.30289600
Co	0.55775500	1.41194500	-0.10087600
Co	-1.68444300	0.24918600	-0.90752700
Co	-1.07770100	-0.06870900	1.40793500
N	2.31031700	-0.49537300	0.10545000
N	2.33101000	0.84818900	0.22496600
H	1.41710200	-1.03568800	1.01040700
H	3.20446400	-0.97389900	0.07696000

 This journal is © The Royal Society of Chemistry 20xx

H	3.24932000	1.24879300	0.06374400
H	0.77300800	2.84611100	-0.62106900

TS5-6

Rh	-0.22576900	-0.97438400	0.66268700
Co	0.28033200	0.83553300	-1.09582100
Co	-2.01063100	0.41229900	-0.29755900
Co	1.10893600	1.11844200	1.14192600
N	1.24092700	-1.68589500	-0.91646800
N	1.57649200	-0.52401600	-1.71640400
H	2.06049200	-2.08536800	-0.46867400
H	0.83278200	-2.38579000	-1.52632200
H	1.77817700	0.46963600	-0.58846700
H	2.54304000	-0.57122500	-2.01809500

TS6-7

Rh	-1.16972300	0.41639800	1.14603500
Co	-2.24372600	2.08395200	-0.61643300
Co	0.23187000	1.85006000	-0.31157900
Co	-1.39567200	-0.07417300	-1.29898800
N	-2.89257300	1.25383300	1.99088000
N	-3.77567800	2.00615600	0.64638400
H	-3.57589600	0.71276900	2.52203900
H	-2.66375800	2.07958800	2.53576800
H	-4.40514000	1.24824500	0.39831700
H	-4.34289000	2.76104900	1.03463900

TS8-9

Rh	-2.12952800	0.25474000	1.78897500
Co	-2.88123300	1.35426100	-0.31704500
Co	-0.33527100	0.95657100	0.03896600
Co	-1.89083600	-1.00141500	-0.33479300
N	0.91404700	1.33900200	1.43821900
N	-4.50327200	2.17993200	-0.53256600
H	0.94664400	2.18744400	1.98967400
H	1.56116500	0.66816400	1.83505600
H	-4.63366800	3.03164700	-1.06230900
H	-5.32407400	2.01535100	0.03228600
H	-0.49017600	0.71566300	1.87450000
H	-2.31804900	-1.38632000	1.36356900

TS10-11

Rh	-0.26461900	0.62366500	-1.00209300
Co	0.86139200	-1.03179500	0.47053400
Co	0.29835500	1.32108300	1.18326100
Co	-1.59682900	-0.99356600	0.31599300
N	2.38984900	-0.99347100	-0.69118100
H	2.59421300	-1.68441000	-1.40223800
H	3.11896300	-0.29464200	-0.66980200
H	1.26695400	-0.11608600	-1.17979100

Quintet reaction system

$RhCo_3$

Rh	0.92400200	0.71801600	0.23622500
Co	0.52910100	-1.49486100	-0.50370900

Co	-1.02749300	-0.30032700	1.22549500
Co	-1.04161300	0.59849300	-1.11549400

H_2RhCo_3

Rh	-1.19540200	0.06305300	0.36287000
Co	1.23927000	0.04442000	1.14119500
Co	0.43259600	-1.33092500	-0.76436500
Co	0.50336000	1.20047900	-0.93501800
H	-2.18920900	-0.49912700	-0.87145300
H	-2.74881200	-0.01554100	-0.38664900

IM1

Rh	-1.26295900	0.66428700	0.75746700
Co	-2.66561700	1.52017100	-1.11604000
Co	-0.21381800	1.55162200	-1.26800100
Co	-1.37377900	-0.57615600	-1.50541600
N	-2.62958200	2.06944600	1.59545700
N	-3.24905700	2.31478900	0.66571500
H	0.13488300	0.16664500	1.60886800
H	-0.28787300	0.63251900	2.20290600

IM2

Rh	-1.75048200	0.38037400	1.12341100
Co	-2.45795600	1.75306000	-0.81719900
Co	0.11679600	1.12013800	-0.34733400
Co	-1.41130900	-0.35426200	-1.51047700
N	-3.19148000	1.74027100	1.57910000
N	-3.47954700	2.42127700	0.58032800
H	-0.10301700	0.79001600	1.39032600
H	-3.50818300	2.11026400	2.48501700

IM3

Rh	-1.82613000	0.44601800	1.23911600
Co	-2.65589400	1.88905200	-0.58994000
Co	-0.04929300	1.33498200	-0.25249500
Co	-1.54694800	-0.13245700	-1.45421800
N	-3.22667500	1.74487300	1.85278500
N	-3.59502800	2.53354100	0.97722200
H	-0.20232300	0.95109000	1.47707900
H	-3.49061700	1.98032600	2.82151900
H	-3.26938400	2.44890900	-2.12391000
H	-3.77070500	2.77608800	-1.60435200

IM4

Rh	-1.53319400	1.07808500	1.33870400
Co	-2.46637100	2.07399400	-0.80015400
Co	0.06117400	1.32930100	-0.49378500
Co	-1.57340600	-0.51484400	-0.44439700
N	-3.44940200	1.82952400	1.55375900
N	-3.85427400	2.37860300	0.40477100
H	0.09714700	0.77775000	1.25573100
H	-3.96499000	2.14179500	2.37241200
H	-2.64503200	3.20021200	-1.83080900
H	-4.52736200	3.13486700	0.49818200

This journal is © The Royal Society of Chemistry 20xx

IM5

Rh	-1.54635600	1.12168000	1.37927300
Co	-2.50002800	2.08303800	-0.72157900
Co	0.02754000	1.31900800	-0.56540900
Co	-1.62533800	-0.40350100	-0.41545000
N	-3.51292300	1.86738400	1.70629000
N	-3.94485100	2.40246400	0.40646900
H	-3.53930000	2.57429300	2.43665700
H	-4.15573100	1.12859500	1.96720600
H	-2.88603900	3.03404000	-1.86498200
H	-4.09280200	3.40132300	0.53462500

IM6

Rh	-0.28774200	-0.97569000	-0.46326900
Co	-0.44921900	1.39029100	0.19082000
Co	1.00295000	-0.35299800	1.44563900
Co	1.64837400	0.48951100	-0.90530900
N	-2.42724200	-0.56935200	0.12046400
N	-2.55841800	0.88298800	-0.01826600
H	-3.28079300	-1.03916600	-0.17228200
H	-2.26294700	-0.76024600	1.10456700
H	-2.67504700	1.06652900	-1.00955200
H	-3.39006400	1.21975400	0.46795300

IM7

Rh	-1.48924300	0.00505600	1.42852700
Co	-3.06895700	1.46919100	0.05263200
Co	-0.62043300	1.65262400	-0.01156000
Co	-1.66336500	-0.28072000	-1.02615000
N	0.44826400	1.01353900	1.46949400
N	-4.48918700	2.33597500	-0.70283400
H	0.64170100	1.58105900	2.28592300
H	1.21415700	0.36738900	1.32770700
H	-4.81482200	2.20186800	-1.64949100
H	-4.88531100	3.19235300	-0.33843900

IM8

Rh	-1.32451700	-0.27067600	1.62697000
Co	-3.08675900	1.05723000	0.37269600
Co	-0.90847600	1.64397200	-0.17363000
Co	-1.65459200	-0.58072300	-0.87884400
N	0.21975800	1.18284700	1.33494300
N	-4.22114600	2.18727600	-0.46550000
H	0.32466500	1.80765400	2.12672300
H	1.12313300	0.76197800	1.14736400
H	-4.17971600	2.41836600	-1.44784100
H	-4.72324900	2.91818100	0.02350200
H	-2.25768100	-1.62674700	1.74659900
H	-2.80099300	-0.91216500	1.97988600

IM9

Rh	-1.77452600	-0.39602900	1.43517500
Co	-2.75975200	1.19902500	-0.12927000
Co	-0.40941800	0.41005900	-0.59836800
Co	-2.21984000	-1.17602800	-0.88511700

N	1.06889400	1.30808600	-1.75418900
N	-3.97980900	2.55710000	-0.02645900
H	0.60969600	1.99691100	-2.34271800
H	1.54117600	0.65167300	-2.36928300
H	-3.87749200	3.31529600	0.63568300
H	-4.96853100	2.36012500	-0.12469700
H	1.78279400	1.79438900	-1.21876900
H	-2.58161000	-1.71726900	0.84291000

IM10

Rh	-0.92396500	0.53100800	1.17375000
Co	-2.64439600	1.37050200	-0.40630600
Co	-0.76282900	0.01429800	-1.34930200
Co	-2.51509800	-1.03706700	0.00272700
N	-4.08215700	2.40696500	0.00041500
H	-4.13159100	3.37351000	-0.29564800
H	-4.59668900	2.30340100	0.86317700
H	-2.01446600	-0.64653000	1.68463500

IM11

Rh	-0.83945600	0.05414600	1.00498200
Co	1.37225300	-0.15909700	0.04125500
Co	-0.73661000	-1.35961600	-0.87066900
Co	-0.53972600	1.37916800	-0.91138500
N	3.40217000	0.05471900	0.17072700
H	3.89473700	-0.83179600	0.22199800
H	3.79062200	0.59006400	-0.59993100
H	3.59355100	0.56500200	1.02686300

TS1-2

Rh	-1.03656700	0.35173300	0.84889900
Co	-2.41392900	1.81066800	-0.83170200
Co	0.21846100	1.05391400	-1.10820900
Co	-1.74513200	-0.39395600	-1.51044700
N	-2.45889400	1.39391400	1.50269200
N	-3.15408000	2.28400300	1.15541000
H	0.69369800	0.42715400	0.50677100
H	-1.65135800	1.41589400	2.37754200

TS3-4

Rh	-1.88974100	0.54519000	1.09288500
Co	-2.39587000	1.62167300	-1.12491100
Co	0.03264000	1.25423200	-0.38912700
Co	-1.33579700	-0.59172200	-1.20766600
N	-3.37176700	1.83750000	1.65816500
N	-4.02391900	2.74888500	1.20241900
H	-0.36779000	1.31330600	1.32454500
H	-3.51741000	1.65821800	2.68089400
H	-3.49201800	2.90270100	-1.16051600
H	-3.67763100	2.82551300	-0.24460500

TS4-5

Rh	-1.95216700	0.62278300	1.24961400
Co	-2.69965200	1.79123300	-0.82251300
Co	-0.26757800	1.81485500	-0.11755500

 This journal is © The Royal Society of Chemistry 20xx

Co	-0.98685300	-0.35090900	-0.89435100
N	-3.40239700	2.00568300	1.66452300
N	-3.81062600	2.55139100	0.49770000
H	-2.08731500	2.17878700	1.87199200
H	-3.82769500	2.39293500	2.50210700
H	-2.92965100	2.76524600	-1.97640300
H	-4.17363600	3.49700000	0.58741600

TS5-6

Rh	-1.72250800	0.61452900	1.22947200
Co	-2.13062100	2.31461800	-0.65424400
Co	0.15182900	1.71652000	-0.04908600
Co	-2.62493500	-0.00655300	-1.04821500
N	-3.45787600	2.00293000	1.70226600
N	-3.72350700	2.67952600	0.42746000
H	-3.17289400	2.68161000	2.40268600
H	-4.25423100	1.46011300	2.03231600
H	-3.62948700	1.50241300	-0.52578800
H	-4.67644300	3.03573100	0.36665100

TS6-7

Rh	-1.16987500	0.46441000	1.17505400
Co	-2.24060700	2.08911400	-0.60507300
Co	0.26049900	1.76781000	-0.37873700
Co	-1.36559100	-0.03116500	-1.33995300
N	-2.91047900	1.26931300	2.00762300
N	-3.76962600	1.99620400	0.66257000
H	-3.58908400	0.70631600	2.52118100
H	-2.70743100	2.09190500	2.56730700
H	-4.39085600	1.23577600	0.39946100
H	-4.35013500	2.74819300	1.03762900

TS8-9

Rh	-1.49027800	-0.84497800	1.46413100
Co	-3.09607200	0.90039700	0.54586000
Co	-0.49189100	1.07153200	0.26829000
Co	-1.77708600	-0.44677200	-1.10712900
N	0.43549500	1.73849700	1.74309600
N	-3.86426200	2.39561400	-0.17255200
H	0.04467900	2.37976400	2.42013400
H	1.33851000	1.40878100	2.05560700
H	-3.51052500	2.94145000	-0.94321800
H	-4.80538000	2.69645600	0.04337800
H	-0.50078100	0.45250200	1.97389500
H	-2.94308800	-0.24353000	1.99330400

TS10-11

Rh	-1.21153200	0.34833300	1.01862500
Co	-3.20403100	0.45457600	-0.31022500
Co	-0.92448900	0.52579000	-1.40818500
Co	-1.90363600	-1.57427500	-0.64040700
N	-3.86159700	2.01844100	0.48167000
H	-3.61482900	2.95408000	0.19003500
H	-4.41191400	2.05287300	1.32979100
H	-2.53916500	1.53626800	1.01214400

Septet reaction system

$RhCo_3$

Rh	-1.07367400	-0.11651000	-0.17198700
Co	0.87702300	0.04289500	-1.47123400
Co	0.35748300	1.44750600	0.83735200
Co	0.64010700	-1.28937900	0.92521900

H_2RhCo_3

Rh	1.19999200	0.24760300	0.29116300
Co	-1.24152700	0.41572000	1.06814500
Co	-0.60026400	0.74440100	-1.27422000
Co	-0.33961000	-1.54665100	-0.23652100
H	2.16614900	-0.66427400	-0.72571300
H	2.73204200	-0.04152300	-0.42654700

IM1

Rh	-1.26233300	0.67151800	0.75207600
Co	-2.69242900	1.49136900	-1.13077600
Co	-0.18083400	1.54093900	-1.25941700
Co	-1.38195500	-0.56958000	-1.51499400
N	-2.60586500	2.08599900	1.58200100
N	-3.27567700	2.33099000	0.68826500
H	0.16516000	0.22449400	1.60023400
H	-0.31386900	0.56759500	2.22356600

IM2

Rh	-1.77461900	0.33670900	0.97626100
Co	-2.56348500	1.70801700	-0.83610200
Co	0.16820400	1.03764900	-0.28566600
Co	-1.38449500	-0.33953400	-1.59010000
N	-3.05069200	1.76751700	1.58135700
N	-3.47986000	2.40209700	0.59748000
H	-0.34742800	0.96856200	1.53028100
H	-3.35280300	2.08012100	2.50966200

IM3

Rh	-1.72200300	0.45910800	1.21282900
Co	-2.76174600	1.67768900	-0.61149400
Co	-0.22812400	1.32711000	-0.59765300
Co	-1.68202400	-0.62963500	-1.09437400
N	-3.14942800	1.68289000	1.88591500
N	-3.77999000	2.19566300	0.96741300
H	-0.52047800	1.62459200	1.17726200
H	-3.50694400	1.82258500	2.84251800
H	-2.92255400	2.72576800	-1.99795400
H	-3.35970600	3.08665100	-1.44165700

IM4

Rh	-1.59164900	1.20852200	1.26794700
Co	-2.56225800	2.49638700	-0.67902400
Co	-0.40963800	1.48985000	-0.97488900
Co	-1.58381000	-0.61349300	-0.21628400
N	-3.54160000	1.74894200	1.65018800
N	-3.93169600	2.51378700	0.61745300

This journal is © The Royal Society of Chemistry 20xx

H	0.01333800	1.12351000	0.79303600
H	-4.16302200	1.80538300	2.45368400
H	-1.34448100	2.56044200	-1.87736300
H	-4.74089400	3.09595700	0.81966600

IM5

Rh	-1.63319500	1.47354700	1.28172100
Co	-2.97406700	1.76254900	-0.87356600
Co	-0.16363900	1.78107400	-0.62118500
Co	-1.57542800	-0.26685100	-0.48562600
N	-3.73521200	1.79568000	2.10508900
N	-3.36408100	2.58075100	0.95531500
H	-3.93645200	2.36405700	2.91847300
H	-4.52708900	1.20060100	1.89958100
H	-3.63482900	1.64618000	-2.29363500
H	-3.34308400	3.56316300	1.20465100

IM6

Rh	-0.27390800	-0.95322300	-0.52576700
Co	-0.43720400	1.41058500	0.11229000
Co	0.88942000	-0.36494400	1.46363700
Co	1.70148000	0.47157800	-0.85860200
N	-2.41794500	-0.56896400	0.11902900
N	-2.56214500	0.88108600	-0.01325500
H	-3.28164100	-1.04361300	-0.13340000
H	-2.20497000	-0.75945600	1.09426500
H	-2.71543500	1.06501100	-0.99941100
H	-3.37780100	1.21356100	0.50197700

IM7

Rh	-1.36276200	-0.30642200	1.27587900
Co	-2.99537600	1.22074600	0.16746100
Co	-0.66668800	1.40018200	-0.38702200
Co	-1.75871400	-0.73288300	-1.13562800
N	0.16310200	1.18224900	1.37835900
N	-4.21240100	2.39208100	-0.54193900
H	0.04181600	1.89373800	2.09049600
H	1.08899400	0.78289800	1.48809900
H	-4.27224300	2.66312800	-1.51270400
H	-4.75292400	3.04261700	0.01280700

IM8

Rh	-1.33719100	-0.25123400	1.59340100
Co	-3.00790400	1.14400600	0.34224000
Co	-0.72228200	1.45250900	-0.33436600
Co	-1.64090700	-0.70222900	-0.90730700
N	0.22529000	1.19932300	1.34610700
N	-4.24858200	2.23899200	-0.43534800
H	0.23392400	1.91080800	2.06808600
H	1.14065100	0.76208200	1.33320700
H	-4.25549600	2.50957300	-1.40781200
H	-4.81845000	2.88846200	0.09080000
H	-2.28946600	-1.61320400	1.64871200
H	-2.76915900	-0.95189400	2.05514900

IM9

This journal is © The Royal Society of Chemistry 20xx

Rh	-1.53903700	-0.49803500	1.48224200
Co	-3.01653600	1.14055100	0.40249100
Co	-0.24175900	0.37160300	-0.30569800
Co	-2.39914600	-0.82948700	-0.84513200
N	1.01537700	1.28081400	-1.65911600
N	-4.23410400	2.46949700	0.03982000
H	1.10440000	2.27217600	-1.45680100
H	0.62785500	1.18923300	-2.59346400
H	-3.99487200	3.44765900	0.14411700
H	-4.90685500	2.38276300	-0.71014900
H	1.94615600	0.87536000	-1.66318100
H	-1.92989800	-1.79879700	0.62976900

IM10

Rh	-0.82395200	0.31407500	1.11315400
Co	-2.53188100	1.58140700	-0.09539100
Co	-0.95325500	0.17343000	-1.43032900
Co	-2.50829700	-1.05753800	0.00214300
N	-3.40627300	3.05144700	-0.74960100
H	-3.14354300	3.59858300	-1.55551300
H	-4.26992800	3.41341600	-0.36882800
H	-2.03376600	-0.59545500	1.74436800

IM11

Rh	-0.92979500	-0.16347000	0.89321000
Co	1.38398000	-0.03107900	0.39486000
Co	-0.61581200	-1.23071500	-1.15892400
Co	-0.67975400	1.48856900	-0.73688600
N	3.42554100	0.04749200	0.19579300
H	3.84634300	-0.82129300	0.51080700
H	3.69923500	0.19938400	-0.77038200
H	3.80780300	0.80370200	0.75536300

TS1-2

Rh	-1.12343300	0.28412700	0.74035800
Co	-2.42104400	1.76299700	-0.69341600
Co	0.25845400	1.06321900	-1.08135700
Co	-1.68392700	-0.30398100	-1.72735300
N	-2.56576600	1.42039700	1.91394300
N	-3.06431000	2.08490100	1.01189800
H	0.54102800	0.45372500	0.62841500
H	-1.48880500	1.57793800	2.14846700

TS3-4

Rh	-1.93016600	0.52342300	1.02299000
Co	-2.45049100	1.65059900	-1.03271300
Co	0.11941900	1.18161600	-0.28471600
Co	-1.29260000	-0.51372900	-1.33942500
N	-3.30520100	1.83826000	1.71196900
N	-3.89916000	2.76641000	1.19625600
H	-0.46515800	1.31766100	1.38367400
H	-3.49575300	1.71539400	2.72785400
H	-3.62797100	2.82784200	-1.25383500
H	-3.69222100	2.80802100	-0.29997000

TS4-5

 This journal is © The Royal Society of Chemistry 20xx

Rh	-1.74893700	1.84584500	1.50467100
Co	-3.00663100	1.77173000	-0.76384200
Co	-0.40900200	1.81283300	-0.64740800
Co	-1.54030100	-0.20814000	0.23206500
N	-3.80861400	2.28826400	2.10624300
N	-3.55344000	2.84953400	0.86639900
H	-2.69961200	1.61588400	2.84107400
H	-4.27675100	1.40212800	1.92863900
H	-3.55150100	1.30908200	-2.15644400
H	-3.40173600	3.84379000	1.00482200

TS5-6

Rh	-1.92776000	0.50348800	1.14538700
Co	-2.44508300	1.98289300	-0.68854800
Co	-0.05280400	1.55454100	0.05848500
Co	-1.28228900	-0.21494900	-1.14892200
N	-3.42477100	2.02550000	1.77394400
N	-3.71838900	2.75683700	0.54762600
H	-4.26957400	1.66526000	2.20631800
H	-2.97183600	2.66525800	2.41766900
H	-3.98823100	1.81663400	-0.55132800
H	-4.68052300	3.07470600	0.55266800

TS6-7

Rh	-0.28378600	-1.14538200	-0.13765100
Co	-0.49683500	1.30174300	-0.23111700
Co	0.99339100	0.11500600	1.49418800
Co	1.69996100	0.29113700	-0.85917900
N	-2.27373300	-0.82234000	0.20990400
N	-2.41729000	0.91167500	0.05567300
H	-2.99353100	-1.15983000	-0.42177400
H	-2.58730100	-0.98041400	1.16114400
H	-3.08820200	1.02274700	-0.70282300
H	-2.89398000	1.19057600	0.91143300

TS8-9

Rh	-1.46953100	-0.79074400	1.49092000
Co	-3.10388000	0.89880900	0.58455500
Co	-0.31629200	1.07525700	0.11219800
Co	-1.80117700	-0.41935800	-1.05546400
N	0.45219000	1.69939000	1.74440400
N	-3.86103100	2.39828800	-0.13608500
H	0.06101400	2.44126900	2.31130900
H	1.29498300	1.35188100	2.18171500
H	-3.53219400	2.90474600	-0.94383700
H	-4.79349300	2.71450000	0.09533300
H	-0.58802900	0.54340700	1.96887900
H	-3.00324000	-0.36773300	1.93086900

TS10-11

Rh	-1.09180000	0.57757900	0.93460200
Co	-2.84174300	0.97148900	-0.76354100
Co	-0.74489500	0.09162500	-1.55857900
Co	-2.19473900	-1.31284700	-0.11478200
N	-3.76676500	2.02172300	0.56578700

H	-3.70229100	3.02620400	0.67274900
H	-4.45555100	1.65500500	1.20861200
H	-2.52614400	1.47656700	1.11201600

This journal is © The Royal Society of Chemistry 20xx
